# Supplementary material for: End Sequence Analysis Toolkit (ESAT) expands the extractable information from single-cell RNA-seq data
Source: Genome Res. 2016 Oct;26(10):1397–410. doi: 10.1101/gr.207902.116 (PMC5052061; doi:10.1101/gr.207902.116)
Supplement: Supplemental Material [file supp_gr.207902.116_Supp_Materials.tar.gz › ESAT-esat_v0.1/doc/html/classjaligner_1_1formats_1_1_format_factory.html]

ESAT: jaligner.formats.FormatFactory Class Reference


ESAT


- Main Page
- Related Pages
- Packages
- Classes
- Files

- Class List
- Class Index
- Class Hierarchy
- Class Members

All Classes Namespaces Files Functions Variables Enumerator Pages

- jaligner
- formats
- FormatFactory

Public Member Functions |
Static Public Member Functions |
List of all members

jaligner.formats.FormatFactory Class Reference

|  |  |
| --- | --- |
| Public Member Functions | |
| void | registerFormat (Format format) |
|  | |
| Format | getFormat (String id) |
|  | |
| Collection | getFormats () |
|  | |

|  |  |
| --- | --- |
| Static Public Member Functions | |
| static FormatFactory | getInstance () |
|  | |

## Detailed Description

Formats factory.

Author
:   Ahmed Moustafa (ahmed.nosp@m.@use.nosp@m.rs.sf.nosp@m..net)

## Member Function Documentation

|  |  |  |  |  |  |
| --- | --- | --- | --- | --- | --- |
| Format jaligner.formats.FormatFactory.getFormat | ( | String | *id* | ) |  |

Returns an instance of Format.

Parameters
:   |  |  |
    | --- | --- |
    | id | format id |

Returns
:   Format or null if id not found

|  |  |  |  |  |
| --- | --- | --- | --- | --- |
| Collection jaligner.formats.FormatFactory.getFormats | ( |  | ) |  |

Returns a list of registered formats

Returns
:   Collection

|  |  |  |  |  |
| --- | --- | --- | --- | --- |
| static FormatFactory jaligner.formats.FormatFactory.getInstance | ( |  | ) |  |

static

Returns an instance for FormatFactory.

Returns
:   FormatFactory

|  |  |  |  |  |  |
| --- | --- | --- | --- | --- | --- |
| void jaligner.formats.FormatFactory.registerFormat | ( | Format | *format* | ) |  |

Registers format.

Parameters
:   |  |  |
    | --- | --- |
    | format | instance of format |

---

The documentation for this class was generated from the following file:

- java/jaligner/formats/FormatFactory.java


---

Generated on Fri Aug 15 2014 15:11:06 for ESAT by  

 1.8.7
